# A Semi-Automatic Image-Based Close Range 3D Modeling Pipeline Using a Multi-Camera Configuration

**DOI:** 10.3390/s120811271

**Published:** 2012-08-14

**Authors:** Jiann-Yeou Rau, Po-Chia Yeh

**Affiliations:** Department of Geomatics, National Cheng-Kung University, No.1, University Road, Tainan 701, Taiwan; E-Mail: ypcsogoodshot@gmail.com

**Keywords:** image-based 3D modeling, multi-image matching, multi-camera framework

## Abstract

The generation of photo-realistic 3D models is an important task for digital recording of cultural heritage objects. This study proposes an image-based 3D modeling pipeline which takes advantage of a multi-camera configuration and multi-image matching technique that does not require any markers on or around the object. Multiple digital single lens reflex (DSLR) cameras are adopted and fixed with invariant relative orientations. Instead of photo-triangulation after image acquisition, calibration is performed to estimate the exterior orientation parameters of the multi-camera configuration which can be processed fully automatically using coded targets. The calibrated orientation parameters of all cameras are applied to images taken using the same camera configuration. This means that when performing multi-image matching for surface point cloud generation, the orientation parameters will remain the same as the calibrated results, even when the target has changed. Base on this invariant character, the whole 3D modeling pipeline can be performed completely automatically, once the whole system has been calibrated and the software was seamlessly integrated. Several experiments were conducted to prove the feasibility of the proposed system. Images observed include that of a human being, eight Buddhist statues, and a stone sculpture. The results for the stone sculpture, obtained with several multi-camera configurations were compared with a reference model acquired by an ATOS-I 2M active scanner. The best result has an absolute accuracy of 0.26 mm and a relative accuracy of 1:17,333. It demonstrates the feasibility of the proposed low-cost image-based 3D modeling pipeline and its applicability to a large quantity of antiques stored in a museum.

## 1. Introduction

The generation of a photo-realistic 3D model of close range objects is an important task for cultural heritage documentation [1–5], human face and torso modeling [6,7], industrial reverse engineering [8], *etc*. Since 1885 close range photogrammetry has been the method most often adopted for cultural heritage documentation [9]. Recently, advances in laser scanner technology have created much interest in the utilization of terrestrial laser scanning (TLS) for heritage documentation [10]. Although TLS can obtain high levels of geometric detail with high degrees of accuracy, at the stages of data acquisition and data processing, intensive labor, experience and time are still needed [2]. Meanwhile, the TLS data cannot by itself show textural information. Integration and registration with digital camera images is necessary [2,11]. This increases not only the cost, but also the complexity of the data processing pipeline. It turns out that image-based modeling is the most economical, flexible, portable, and widely used approach for terrestrial 3D modeling [12]. For example, it is popularly adopted not only for close range applications [13], but also for airborne [14] and spaceborne imagery [15]. It has many advantages compared to laser scanning, such as fast data acquisition, abundant texture information, knowledge of the measured locations, and measurement can be performed any time, even when the target has been destroyed [8]. The most important of these is the texture from which you can reconstruct a photo-realistic 3D model or a real 3D model suitable for further scientific study [3,13].

Comprehensive comparisons of current technologies for terrestrial 3D modeling, including image-based rendering (IBR), image-based modeling (IBM), and range-based modeling, can be found in the literature [2,7]. However, there is no single modeling technique which can satisfy the requirements for diverse close range applications in terms of geometric accuracy, level of detail, degree of automation, portability, flexibility, photo-realism, cost and efficiency [16]. Thus, the integration of multiple techniques and data sources is recommended, particularly for the modeling of large-scale heritage sites [17].

In order to accomplish the goal of accurate image-based 3D modeling, the interior and exterior orientation parameters of the image are indispensable. For close range 3D modeling, the relative orientation is sufficient. However, the accuracy of the generated 3D geometric model is directly dependent on the accuracy of the relative orientation parameters (ROPs). The photo-triangulation results derived from bundle adjustment are adopted in most cases, but strong imaging geometry with high redundancy, good distribution and accurate tie-point image coordinates is also required. Moreover, for quality assurance purposes a well-trained professional operator is needed, which will decrease the degree of applicability when large quantities of objects are treated. The availability of automatic, reliable and accurate image matching tools can increase the efficiency of photo-triangulation and surface modeling [18,19]. Although fully automatic photo-triangulation schemes have been introduced in the field of computer vision [20–23], they are not yet as reliable and precise as required in photogrammetric 3D modeling. In the meantime, in some situations the on-site environment or poor texture of the object surface may prohibit well distributed tie-point image coordinate measurements as well as obtaining strong imaging geometry. The quality assurance after fully automatic photo-triangulation would be difficult, especially when thousands of objects are treated.

For cultural heritage digital recording, particularly for close range recording of objects such as statues, vases, sculptures, and so on, a scaled relative orientation is sufficient, without the need of absolute exterior orientation parameters (EOPs). Thus, this study proposes a multi-camera framework with invariant ROPs which were obtained through an efficient and accurate calibration procedure. The derived EOPs are further used in multi-image matching for surface point cloud generation. Multiple cameras/images are used to increase redundant measurements for image matching and to avoid ambiguity by means of epipolar constraints. In order to complete the modeling of a large object, multiple stations are required and surface point cloud stitching is necessary [24,25].

In the literature related to camera calibration methods, camera model, target types, calibration field types, and so on, have been reported in the fields of photogrammetry and computer vision. A comprehensive comparison and review of these methods can be found in [26,27]. Most methods utilize a fixed calibration field with convergent imaging geometry and roll angles changed by ±90∼270 degrees to decouple the correlation between the unknowns. Meanwhile, a well distributed image measurement of conjugate points to the full image frame is required to characterize the radial lens distortion. However, sometimes the camera used is not convenient due to its size, weight, cable connections, or space limitations. All of these can cause difficulty in pointing the camera to acquire suitable calibration images. Moreover, there are many situations where the geometry of the image network will not support effective calculation of the camera's interior orientation parameters (IOPs) through the on-the-job (OTJ) calibration [27]. Thus, in this study, instead of using a fixed calibration field, a rotatable round table is proposed for camera calibration, especially for close range photogrammetric applications.

The aim of this study is to provide an economical approach for antique 3D modelling based on close-range photogrammetric technology through a multi-camera configuration, which would be particularly suited for the 3D modelling of the thousands of antiques of similar size stored in a museum, for example. The major issues to be dealt with in the whole 3D modelling pipeline system are: (1) how to perform quality assurance and obtain accurate interior orientation parameters (IOPs) and ROPs through single and multi-camera calibration, (2) how to obtain accurate and reliable 3D surface models through a multi-image matching technique, and (3) how to determine the most suitable multi-camera configuration to ensure accuracy and completeness. Although the object's surface texture is also important for digital documentation, it can be generated automatically once an accurate 3D surface model has been created through the suggested 3D modelling approach because the relationship between the object and image spaces are known. Thus, texture generation is not within the scope of this study. A detailed description of the proposed multiple cameras configuration for close range 3D modeling will be given in the following sections. A novel camera calibration approach that utilizes a rotatable calibration field is suggested. Several case studies are carried out for accuracy analysis using a reference model to find out which multi-camera configuration can achieve the best accurate results. Finally, remarks and findings will be provided in the conclusion.

## 2. The Proposed Methodology

**Figure 1.**
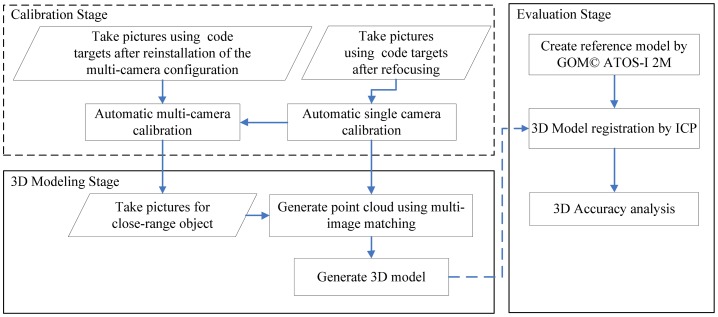
The flow-chart of the proposed 3D modeling pipeline.

There are two stages in the proposed 3D modeling pipeline, as shown in Figure 1. In the calibration stage, the IOPs of each camera are calibrated independently using coded targets through a self-calibration bundle adjustment with additional parameters [28]. This process can be performed fully automatically and should be done when the camera's status has changed. Then, the cameras are installed on a curved metal bar(s) designed to maintain their relative orientation, which is important when dealing with large quantity of objects that require long period of working day. A strong convergent imaging geometry is constructed for better positioning accuracy. The EOPs of all the cameras are again calibrated automatically by means of the coded targets [28]. This procedure needs to be applied every time any camera is reinstalled.

The second stage is 3D modeling of the close range object from images acquired by the same multi-camera configuration. The cameras' calibrated EOPs/IOPs are used for space intersection after multi-image matching to obtain the object's surface point clouds. This means that no photo-triangulation is required for the same multi-camera configuration even if the target is changed and does not require any markers on the object surface. A close range multi-image matching technique is used to fulfill this requirement [18]. The generated point clouds are reorganized as 3D TIN models. An additional evaluation stage is applied to compare our modeling results with a reference model acquired by an active sensor (ATOS-I 2M). Since the whole 3D modeling pipeline can be performed with a high degree of automation, it is very suitable for application in regular operations, such as for the modeling of a large quantity of antiques of similar size stored in a museum. A detailed description of all the steps will be given below.

### 2.1. Single Camera Calibration

#### 2.1.1. The Proposed Approach

**Figure 2.**
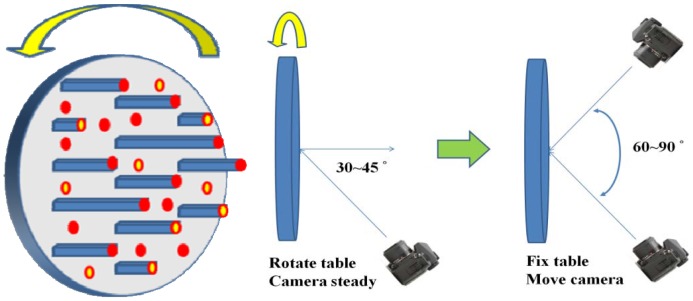
Single camera calibration using a rotatable calibration field.

The purpose of camera calibration is to mathematically describe the internal geometry of the imaging system, particularly after a light ray passes through the camera's perspective center. In order to determine such internal characteristics, a self-calibrating bundle adjustment method with additional parameters is adopted [28,29] that can automatically recognize and measure the image coordinates of retro-reflective coded targets. Based on this functionality, we develop a rotatable round table surmounted by 112 pillars. The coded targets are then fixed to the top of the pillars and the table surface to establish a three-dimensional calibration field with heights varying from 0 to 30 cm. Instead of changing the camera location during image acquisition, the table is simply rotated. Moreover, the camera's viewing direction is inclined 30°∼45° with respect to the table's surface normal. The concept for the acquisition of convergent geometry by means of a rotatable calibration field is illustrated in Figure 2. The round table is rotated at 45° intervals while capturing the calibration images. This results in 8 images with convergent angles of 60° to 90°, which is a strong convergent imaging geometry. In order to decouple the correlation between IOPs and EOPs during least-squares adjustment, it is suggested that an additional eight images be acquired with the camera rotated for portrait orientation, *i.e.*, change roll angle with 90°. Finally, for the purpose of increasing image measurement redundancy, two additional images (landscape and portrait) are taken with the camera's optical axis perpendicular to the table surface.

**Figure 3.**
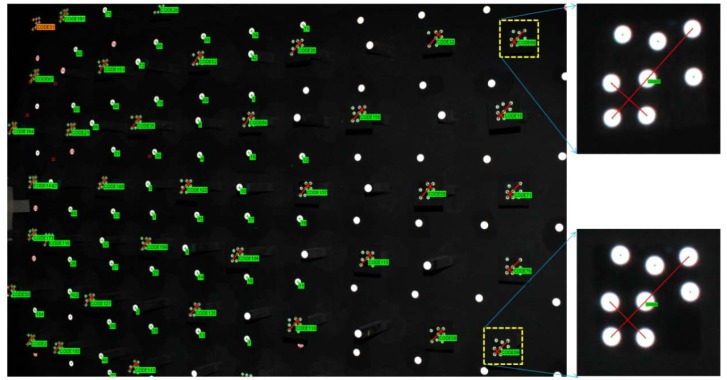
A sample image for camera calibration and two enlarged code targets.

**Figure 4.**
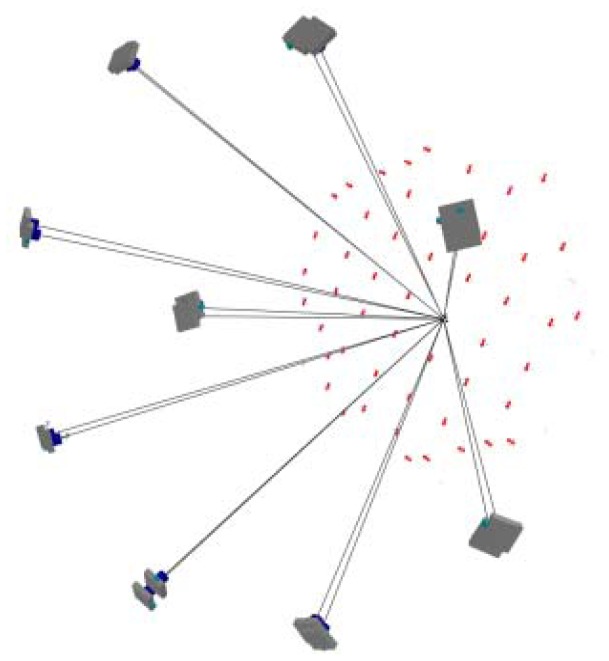
Convergent bundles during camera calibration.

The relative position of all the code targets is firmly fixed and remains stationary during rotation. This is essentially the same as surrounding the calibration field and taking pictures, introducing a ring type convergent imaging geometry. The proposed ring type configuration is difficult to obtain with a fixed calibration field and particularly for a limited space where the floor and ceiling will constrain the camera's location. A sample image for camera calibration is illustrated in Figure 3. One may observe that the coded targets are well spread out to the whole image frame, especially the image corners, where the most critical regions to describe the radial lens distortion are. In the figure, the bigger white dots are designed for low resolution cameras to increase the number of tie-point measurements by the auto-referencing function. The auto-referencing is performed by predicting the detected white dots from one image to the others using epipolar geometry in case the relative orientation has been established in advance by means of code targets. Meanwhile, Figure 3 also shows two enlarged code targets. In each coded target, two distance observables are constructed by four white points and utilized for scaling purposes during bundle adjustment. This means that the absolute positioning accuracy can be estimated for the calibrated IOPs. Figure 4 depicts the result of bundle convergence for one target from all cameras. The results demonstrate that it is possible to obtain a strong imaging geometry by means of the proposed arrangement.

#### 2.1.2. Determination of Additional Parameters (APs)

Two approaches for determining the most significant APs are suggested in this study. The first one is to check the change of square root of the *a posteriori* variance (σ_0_) value, which is a measure of the quality of fit between the observed image coordinates and the predicted image coordinates using the estimated parameters (*i.e.*, image residuals), by adding one additional parameter at a time. The predicted image coordinates consider the collinearity among object point, perspective center, and image point by correcting the lens distortion. Thus, if a significant reduction in σ_0_ was obtained, for example 0.03 pixels, which is the expected accuracy of image coordinate measurement by the automatic centroid determination method [30], the added parameter is considered as significant one because it correct the image coordinates displacement effectively. Otherwise, it can be ignored. This procedure is simpler and easier to understand its geometric meaning when compare with the next approach.

The second approach is based on checking the correlation coefficients among the parameters and the ratio between the estimated value and its standard deviation (σ), namely the significance index (t). If two APs have a high correlation coefficient, e.g., more than 0.9, then the one with a smaller significance index can be ignored. However, if the smaller one is larger than a pre-specified threshold, the added parameter can still be considered significant. The threshold for the significance index is determined experimentally, e.g., based on the results from the first approach.

The significance index (t) is formulated in Equation (1), which is similar to the formula used for stability analysis, as shown in Equation (2), namely change significance (c), used for verifying the stability of a camera's internal geometry. Both formulations are based the Student's test. The significance index (t) is described as:

(1) $$\text{t}=\left|\delta_{\text{i}}\right|/{\text{q}}_{\text{i}}$$

where δ_i_ is the estimated value for parameter i and q_i_ is the standard deviation for parameter i [31], thus t has no unit. The variable t is an index for the null-hypothesis that “the i^th^ AP is not significant” compared to the alternative hypothesis “the i^th^ AP is significant”. On the other hand, the change significance (c) can be described as:

(2) $$\text{c}=\left|\nabla_{\text{i}}\right|/{\text{p}}_{\text{i}}$$

where $p_{i}=\sqrt[{2}]{q_{i,j}+q_{i,j+1}}$; ▽_i_ is the change of parameter i between calibration time j and j + 1 and q_i,j_ is the a-posterior variance of parameter i at camera calibration time j [32], thus c has no unit as well. The variable c is an index for the null-hypothesis that “the i^th^ AP does not change significantly” compared to the alternative hypothesis that “the i^th^ AP changed significantly”.

### 2.2. Multi-Camera Calibration

Many literatures suggest multi-camera configurations for close range 3D modeling. Maas [33] proposed a fully automatic multi-camera calibration procedure by means of a moving reference bar with known length. Heinrichs *et al.* [34] proposed a tri-focal configuration to obtain good intersection angles of epipolar lines.

#### 2.2.1. The Adopted Multi-Camera Configurations

**Figure 5.**
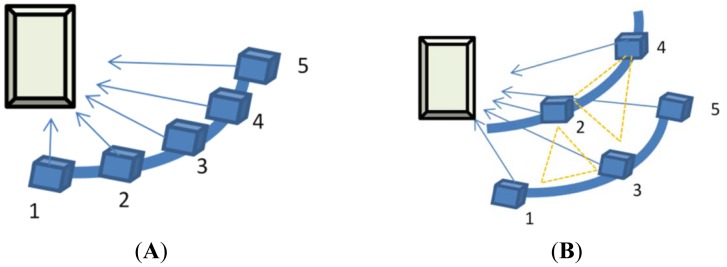
Adopted multi-camera frameworks. (**A**) The 1 × 5 Multi-Camera Configuration. (**B**) The 2 + 3 Multi-Camera Configuration.

The original development of the adopted multiple image matching software is based on conventional stripped aerial images. Thus, in this study two types of multi-camera configurations were proposed, namely the 1 × 5 and 2 + 3 configurations. Figure 5 illustrates the setup of cameras in the proposed multi-camera configurations. In which, the cameras' numbers are denoted and used in the case studies. Several combinations based on those two configurations are compared. In the first setup, five SONY A850 DSLR digital cameras are fixed to a curved metal bar (1.5 meters long), as shown in Figure 5(A), while in the second configuration we use two curved metal bars, as illustrated in Figure 5(B). In the latter case, the lower metal bar has three cameras and the higher one has two. The two metal bars are setup parallel to each other with approximately 30 cm apart. This design is used in multi-image matching to avoid ambiguity problems when searching for candidates along the epipolar line [34]. For better positioning accuracy, the convergent imaging scheme is adopted [19]. For the purpose of synchronous imaging, which is important when the target is a live object, the cameras' triggers are connected in parallel and can be controlled either manually or automatically by a computer. With the 1 × 5 configuration, for an object located at 1.5 meters from the camera, the base-to-depth (B/D) ratios for all camera combinations range from approximately 0.2 to 0.8. The largest B/D ratio will provide accurate space intersection results, whereas the shortest one will introduce less geometric differences which is suitable for area-based image matching. In the experiments, several base-to-depth combinations are tested to evaluate the performance of different multi-camera configurations.

#### 2.2.2. The Proposed Calibration Method

**Figure 6.**
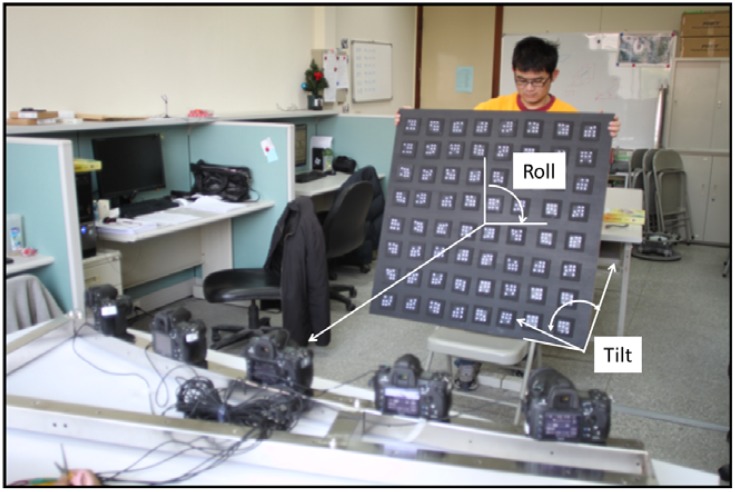
Taking pictures for multi-camera calibration.

For the purpose of multi-camera calibration, the technique with self-calibration bundle adjustment through coded targets was adopted again. Depending on the size of the target, the code targets were uniformly spread throughout an area similar to or larger than the target. Outdoors, the code targets can be spread on the ground. When taking one calibration image dataset, the camera's viewing direction is changed 5∼7 times to construct 90 degrees convergent angle to ensure strong imaging geometry. Acquisition of three more calibration image datasets is suggested by rotating the camera's metal frame for 90, 180 and 270 degrees to increase the redundancy of measurements. For indoor experiments, a portable wooden plate is proposed to fix the code targets and during image acquisition the camera's metal bar can remain stationary. Instead of rotating the metal bar, the wooden plate is inclined at 5∼7 different tilt angles for the construction of a convergent imaging geometry and rotated with 90, 180 and 270 degrees in roll angle. The situation for above mentioned procedure in the laboratory is shown in Figure 6. After automatic recognition of the coded targets, a self-calibration bundle adjustment scheme is utilized to perform photo triangulation and to calculate the EOPs for all images. Under well controls, the five cameras' IOPs can be self-calibrated as well, called on-the-job (OTJ) calibration. However, it is important to make sure that the code targets are well distributed throughout the whole image frame in order to fully characterize the radial lens distortion. Otherwise, it is suggested that the single camera calibration results should be applied and fixed during bundle adjustment for the multi-camera configuration.

#### 2.2.3. Determination of the Exterior Orientation Parameters

**Figure 7.**
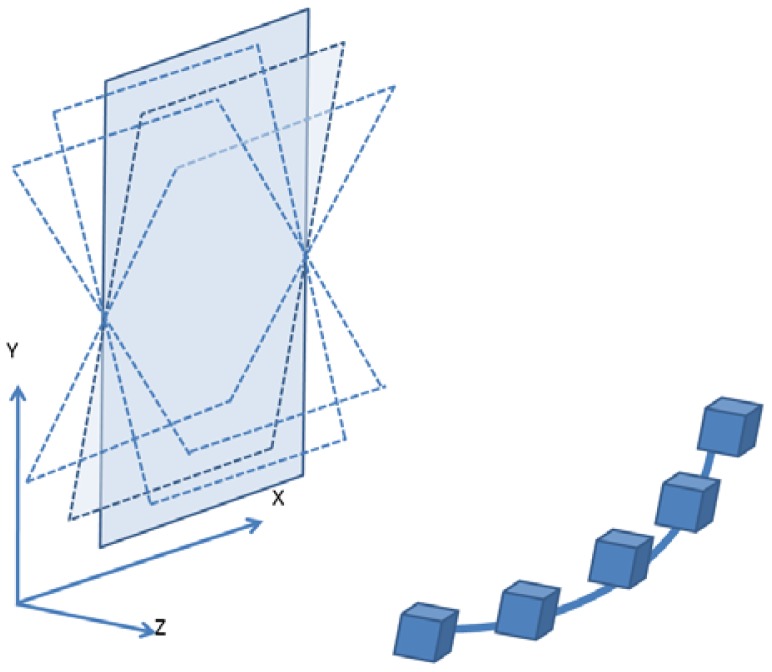
Datum definition and changing of the inclination angle of the wooden plate for the construction of a convergent imaging geometry.

**Figure 8.**
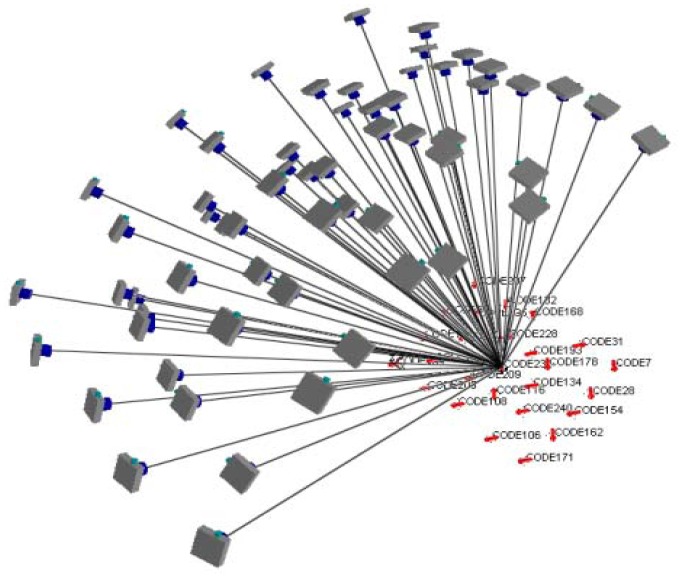
Convergent bundles for the 1 × 5 multi-camera configuration.

Figure 7 illustrates how to define the object space coordinates (datum). The X-axis is parallel to the cameras' alignment orientation; the Y-axis falls on the plane of the wooden plate; and the Z-axis points towards the camera. Generally, the X, Y and Z values of the object space should be shifted as being positive and the EOPs close to vertical imaging are utilized in multi-image matching. This is similar to aerial photography for topographic mapping. Figure 8 depicts the bundle adjustment results and the bundles from one target to all cameras. A strong convergent imaging geometry with high redundancy of image measurement is shown that results in high accuracy and reliable EOPs for further surface modeling. As long as the cameras' relative orientation remains the same, we do not have to redo multi-camera calibration even when photographing different objects or during multi-image matching for point cloud generation. The quality assurance can thus be achieved and remains the same for all targets treated.

### 2.3. Point Cloud Generation by Multi-Image Matching

For surface point cloud generation, several open source or commercial software packages could be used, for example Structure from Motion [35–37], semi-global matching [38], least-squares matching [10,13,16], optimal flow with energy minimization [39], *etc*. A benchmarking evaluation among these algorithms would be interesting to the reader. However, this would be out the scope of this study. Thus, for point cloud generation we adopt a multi-primitive and multi-image matching technique with geometric constraints followed by least-squares area-based image matching to achieve sub-pixel accuracy [10,13,16]. Images with shadows or less texture will be improved by an adaptive smoothing and enhancement filter, *i.e.*, the Wallis filter [40]. For better estimation of the search range during image matching, the object's depth and several seed points can be setup before image matching. The outputs include a rasterized digital surface model (DSM). The generated point cloud for all stereo-pairs can be further edited and for the construction of 3D TINs. It is suggested to perform global registration between them before constructing the 3D TINs in order to eliminate any systematic error come from inaccurate EOPs or IOPs. A detailed description of its functionality can be found in the literature [12,15,18]. There are three image matching strategies distinguished by the number of images, *i.e.*, stereo mode, triplet mode, and multi-image (block) mode. In the block mode, the images can be organized as multiple strips. In the experiments, accuracy analyses of 3D surface modeling for those three modes will be performed to provide the users with an idea of its performance in order to choose a suitable multi-camera configuration.

### 2.4. Accuracy Analysis

The GOM© ATOS-I 2M structure light active sensor is used to acquire an accurate and highly detailed 3D model and applied for accuracy analysis after 3D surface modeling. From the specifications of the device, the created model has an accuracy of better than 0.02 mm, which is suitable for reference to evaluate our modeling results. However, before comparison, the generated model has to be registered with the reference model. The iterative closest point (ICP) surface matching algorithm [25] is used. Finally, a 3D error analysis tool is adopted to evaluate their difference in the object space. Some statistical analysis results will be provided, such as the RMSE, Mean, Maximum and Minimum of the discrepancy. Meanwhile, a pseudo color 3D error model will be provided to facilitate visual inspection of their discrepancies.

## 3. Case Study: A Stone Sculpture 3D Modeling

In this study, performance analyses are carried out for all stages in the proposed scheme. Several close range objects are tested to demonstrate the feasibility of the proposed strategy, including a stone sculpture, one human being and eight Buddhist statues. In camera calibration, the overall accuracy and relative accuracy are adopted. The overall accuracy reflects the absolute 3D positioning accuracy in object space after calibration, while the relative accuracy denotes the ratio between overall accuracy and the maximum range of all targets. The relative accuracy can be used to estimate the overall accuracy when the camera configuration remains the same but the target size is changed.

### 3.1. Single Camera Calibration

#### 3.1.1. Determination of Additional Parameters

A SONY A-850 DSLR camera with SONY SAL50F14 (50 mm) lenses is adopted in this study. The significance test results are summarized in Table 1 and can be examined to illustrate the procedure for determining the most significant APs. In the beginning, all APs are un-fixed during self-calibration bundle adjustment to obtain the correlation coefficients between each other. Compatible with common knowledge, the highest correlation coefficients occurred among K1, K2 and K3, *i.e.*, greater than 0.9. In the first run, we note that the significant indices for K2 and K3 are very low. Since they are highly correlated with K1, they are both ignored. In the second run, K2 and K3 are fixed at zero and the significant indices for P1 and P2 are even lower than for the first run. Thus, they are ignored and fixed at zero at the third run. At the third run, B1 and B2 still have significant indices of 29.7 and 15.8, respectively, which is difficult to determine their significant level. Another approach is thus utilized to determine the significant APs by adding one or two parameters and checking the change of σ_0_ after bundle adjustment. In the lower part of Table 1, one may observe that a significant improvement in the accuracy occurs only when K1 is considered. Even adding K2, K3, P1, P2, B1 and B2 step by step, the σ_0_ has reduced only 0.01 pixels, which is less than the precision of tie-point image coordinate measurement, and the overall accuracy is only improved by 0.0014 mm. This means that they can all be ignored by keeping only the principal distance (c), the principal point coordinates (xp, yp), and the first radial lens distortion coefficient (K1).

#### 3.1.2. Stability Analysis

In practice with terrestrial 3D modeling using DSLR digital cameras, it is sometimes necessary to change the lens or the camera, to reboot the camera, to detach the lens from the same camera, or to refocus for different sizes of object. Thus, in this study, we conduct a series of experiments to analyze the stability of APs of the adopted camera and lens under different situations. Meanwhile, according to previous experiment, only the principal distance (c), the principal point coordinates (xp, yp), and the first radial lens distortion coefficient (K1) are evaluated. The experiments are categorized into three parts.

**Figure 9.**
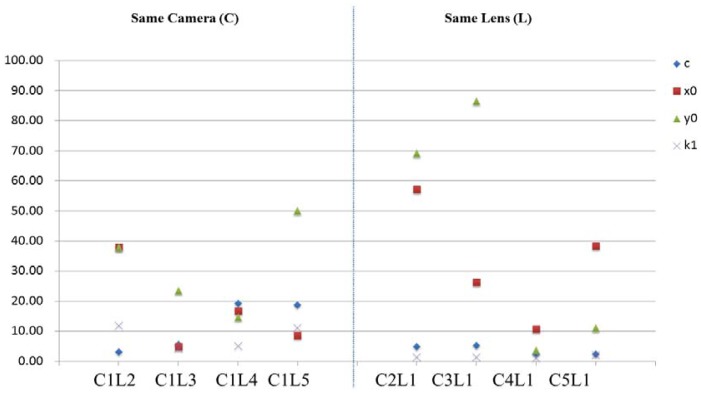
Stability analysis combining five SONY A850 cameras with five 50 mm lenses.

First, we utilize five SONY A850 cameras and five SAL50F14 lenses. Those five lenses are attached on the same camera and calibrated by means of the single camera calibration procedure, respectively. In the second experiment, those five cameras are combined with one of the five lenses and calibrated again using the single camera calibration procedure. The stability analyses are performed using Equation (2) by considering different combinations as different times. The plot of change significance index (c) is illustrated in Figure 9. It is obviously that the internal geometry is unstable when the camera and lens combination is changed. However, for the same lens using different cameras, the principal distance (c) and radial lens distortion (K1) can still be maintained without significant change.

**Figure 10.**
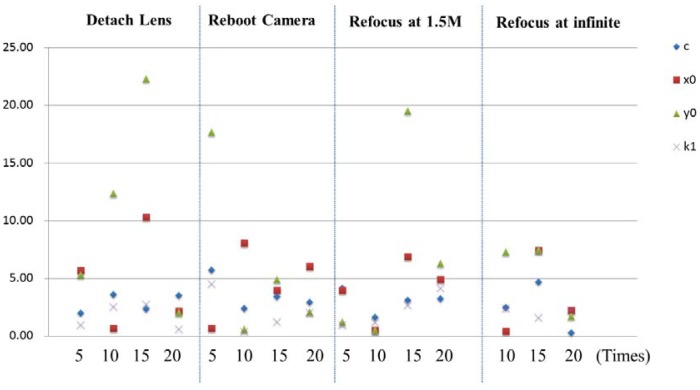
Stability analysis for a SONY A850 camera using a 50 mm lens in different situations.

In the second experiment, single camera calibration is performed after detaching, rebooting, and refocusing (at 1.5 m and infinity) the lens with 0, 5, 10, 15 and 20 times. Since the adopted SAL50F14 lens will perform initialization every time when the camera is rebooted, *i.e.*, the focal distance is reset to infinity. Although, the principal distance is fixed by using a tape to fix the ring on the lens, it is necessary to investigate its stability and its influence on the camera's internal geometry. The change significance plot is shown in Figure 10. It can be seen that the detachment of lens will introduce significant change in its IOPs, particularly for the principal point coordinates. For the task of refocusing and rebooting, for most of the time the APs do not change a lot, but sometimes the principal point coordinates will change significantly. Thus, it is suggested that refocusing or detaching the lens during image acquisition should be avoided. Even rebooting the camera is not recommended, particularly when the lens has initialization functionality.

**Figure 11.**
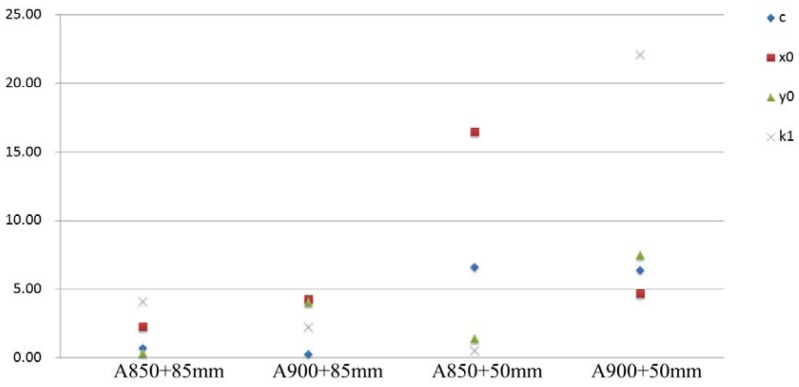
Stability analysis for SONY A850 and A900 cameras using 50 mm and 85 mm lenses.

The purpose of the last experiment is to compare different cameras with different lenses. Here the SONY A850 and A900 cameras with SAL50F14 50 mm and SAL85F28 85 mm lenses are utilized. The SAL85F28 lens can be setup in manual focusing (MF) mode so that no initialization (refocusing at infinite) will be performed after rebooting the camera. There are four combinations obtained by using these two cameras and two lenses. The stability testing is applied for the same camera by comparing the single camera calibration results with the round table fixed or rotated. When the round table is fixed, a 90 degrees convergent angle is constructed from six viewing directions. The camera's roll angles are changed by 0 and ±90 degrees at each location. It results in a total of 18 images for camera calibration. The change significance plot is shown in Figure 11. It can be seen that the 85 mm lens has a manual focusing functionality achieve higher stability in its internal geometry and is independent to the camera used. Thus, it is suggested that a lens with this capability should be considered for high precision 3D modeling purposes.

### 3.2. Accuracy Analysis for Multi-Camera Calibration

Although the SONY SAL50F14 lens is not stable in its IOPs, they are still adopted in the following experiments to evaluate its performance in 3D modeling. Meanwhile, although only two multi-camera configurations are proposed in this paper, *i.e.*, the 1 × 5 and 2 + 3 configurations shown in Figure 5, the term multi-camera can refer to any combination that utilizes more than two cameras.

In this section, the positioning accuracy analyses for seven multi-camera combinations, which include two (C1-C4, C1-C5, C2-C4, C2-C3), three (C2-C3-C4), four (C1∼C4) and five (C1∼C5) cameras in the 1 × 5 configuration with different B/D ratios, are examined. The image acquisition method is described in Section 2.2. The results for the 2 + 3 configuration are similar and thus not discussed. The bundle adjustment for photo-triangulation for each camera combination is conducted independently while their IOPs are all determined by the single camera calibration procedure as discussed in Section 2.1.

After multi-camera calibration for photo-triangulation, among the estimated EOPs of all camera combinations, the mean of standard deviations of all cameras' position and attitude are 0.44 mm and 2.76 × 10^−4^ degrees, respectively, and the their maximums are 0.81 mm and 5.63 × 10^−4^ degrees. Meanwhile, Table 2 summarizes three accuracy indices after photo-triangulation. The overall accuracy is 0.014 mm, the relative accuracy is 1:83,614 and the square root of the a-posterior variance factor (σ_0_) is 0.28 pixels for all cases after multi-camera calibration. These results depict that the estimated EOPs are very close, especially the rotation angles. This means that the proposed multi-camera calibration scheme has good imaging geometry that can achieve very high accuracy and stable results, even when different camera combinations are adopted. This experiment reveals that the change in tilt and roll angles of the calibration board can improve not only the positioning accuracy but also the reliability because high redundancy of image tie-point measurement with strong imaging geometry are obtained.

### 3.3. Accuracy Analysis of 3D Surface Modeling

**Figure 12.**
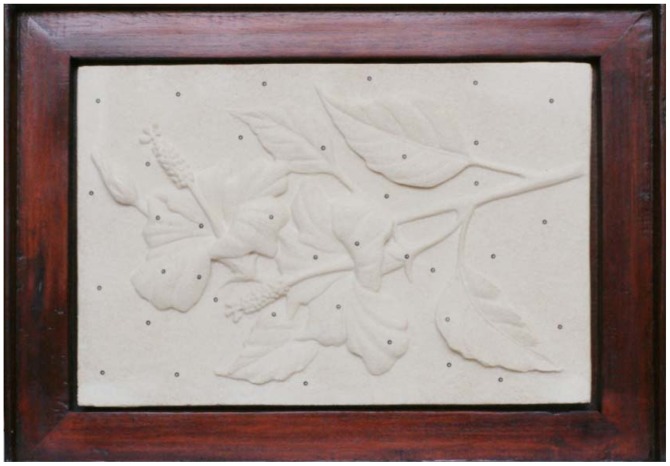
Sample image for 3D modeling (Stone Sculpture).

A stone sculpture with 29 cm × 19 cm in size is used for 3D modeling by both the proposed scheme and the ATOS-I 2M scanner. The sculpture is carved of white sand that gives the surface a homogenous texture which is a challenge to the image matcher. The sculpture is embossed with flowers and leafs giving a relief variation about 10 mm. Figure 12 is an enlarged image from the dataset for 3D modeling which focus on the target only without background and covers about 1/9 of the whole image. Please notice that the sculpture is setup in landscape orientation which is the same as the cameras' orientation. There are several man-made round targets (white dots with black background) attached to the sculpture which are used by ATOS-I 2M for registration purpose, not for the proposed approach.

**Figure 13.**
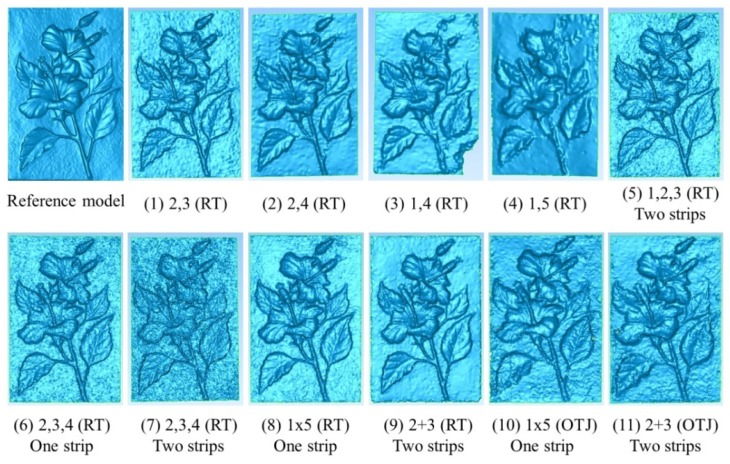
Reference model and all 3D modeling results (stone sculpture).

**Figure 14.**
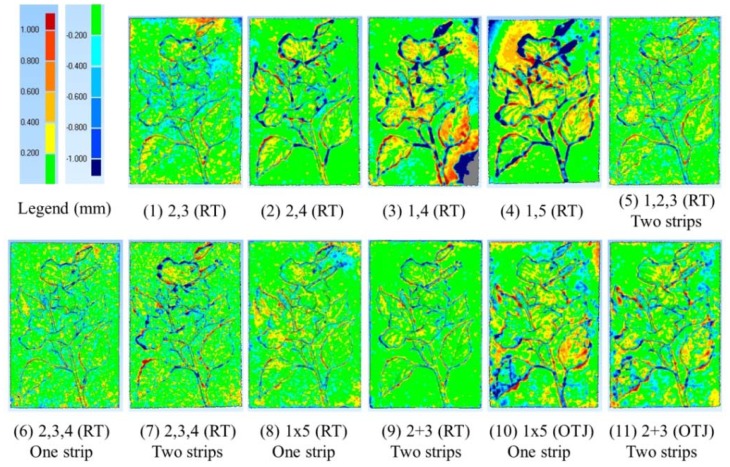
3D error models for all modeling results (stone sculpture).

In this experiment, the modeling results from 11 combinations are compared. In which, two, three and five cameras are arranged at one or two strips with different B/D ratios. Meanwhile, two kinds of IOPs are compared as well; one determined by the proposed single camera calibration method using the round table (RT) and the other one by the on-the-job (OTJ) calibration using images acquired by the multi-camera configuration. The statistics for accuracy analysis are displayed in Table 3. The reference model and the generated 3D models for those 11 case studies are shown in Figure 13. For comparison, the 3D error models are demonstrated in Figure 14. The following issues are discussed by comparing these table and figures.

#### i. B/D ratio

For case numbers 1∼4 with two cameras, the RMSE becomes larger when the B/D is increased. This phenomenon violates the principle of error propagation during space intersection. The reason is clear due to the perspective distortion between the stereo images is high when the B/D ratio gets larger which will introduce more measurement errors in image matching.

#### ii. Y-parallax

By inspecting the 3D error models for case numbers 3 and 4, it should be noticed that large errors occur at the lower-right and upper-right corners of case 3 and upper-left side of case 4. Inspection of the epipolar images shows that these blunders come from large y-parallax. The largest y-parallax is about 8 pixels. Since the IOPs are all the same for cases 1 to 9, this problem could be introduced by inaccurate EOPs. However, from the accuracy analysis after photo-triangulation (case No. 7 in Table 3) it can be seen that the positioning error and σ_0_ are both satisfactory. This phenomenon could be found particularly when high resolution image and only two cameras are utilized. Thus, the adoption of only two cameras for precise 3D modeling is not recommended.

#### iii. Mode in Multi-Image Matching

Inspection of the generated 3D models in Figure 13 shows that the surface is smoother when images with a higher B/D ratio are conducted in the cases with only two cameras (stereo mode), but the errors along the edges of the leafs also get larger by inspecting the corresponding 3D error models shown in Figure 14. As shown in Figure5, the cameras are arranged in normal and inverse triangles (block mode) for case numbers 5 (C1∼C3) and 7 (C2∼C4), respectively. Although the generated 3D models appear to rough, particularly for case number 7, the discrepancy is acceptable as can be seen from Table 3. On the other hand, for case number 6 it is arranged in one strip and the triplet mode, the result has good accuracy with high level of detail. The results get better when the number of cameras is increased to five and using the block mode. This indicates that when the number of cameras is increased the advantage of multi-image matching can be performed; both the range of B/D ratios and the image measurement redundancy can produce higher accuracy and more reliable results.

#### iv. Multiple strips

The purpose for arranging the cameras in multiple strips is to alleviate the ambiguity problem during image matching. However, the accuracy does not improved when three cameras are adopted, *i.e.*, case numbers 6 and 7, but slightly improved for five cameras case, *i.e.*, case number 8 and 9.

#### v. IOPs

**Figure 15.**
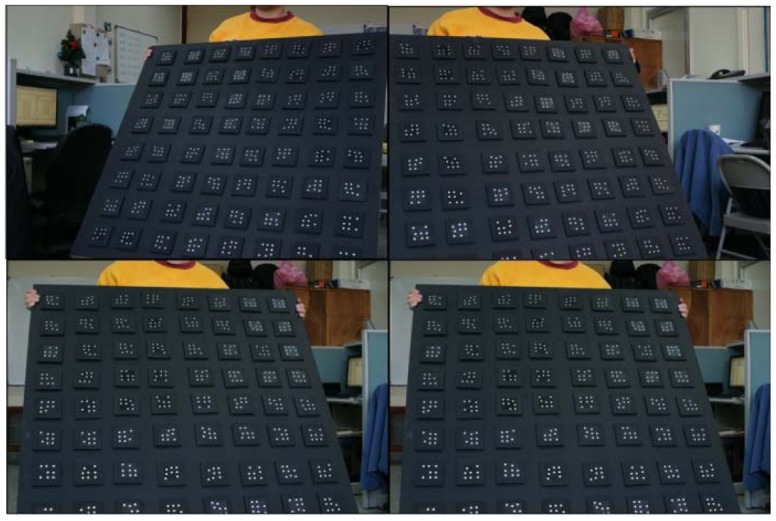
Four calibration image samples from the 1 × 5 configuration.

In cases 10 and 11, the IOPs are determined by the on-the-job (OTJ) approach using images acquired by the multi-camera configuration. The 3D error models and RMSE appear larger when compared with case numbers 8 and 9. This shows that the designed planar surface for on-the-job camera calibration may not properly characterize the camera's internal geometry. Although, the convergent geometry in the proposed multi-camera configuration is strong, the code targets may not be well distributed to the whole image frame particularly when the calibration board is inclined. Four examples from the 1 × 5 configuration are shown in Figure 15 to explain this phenomenon.

#### vi. Computation time in Multi-image Matching

In the proposed 3D modeling pipeline, single camera calibration, multi-camera calibration and 3D TIN generation can all be done in less than five minutes. Since no photo-triangulation is required for different objects, the major bottleneck becomes the time consumed during the multi-image matching. The computation time spent for each case is illustrated in Table 3 and they are all within 35 minutes. It is obvious that more images will take more processing time.

#### vii. Overall

The RMSE for all cases ranged from 0.26∼0.60 mm and the mean of the errors are all close to zero. In the meantime, the maximum and minimum errors are all within ±5 mm. The major discrepancy occurs along the edges of the leafs with a difference of around ±1 mm. The performance analysis results show the best one is case number 10 that utilize five cameras arranged in two strips. The RMSE is 0.26 mm and major detail can be observed in the generated 3D model. Recalling that the relief variation for the stone sculpture is about 10 mm, the obtained accuracy is high without systematic errors during modeling. Comparison of the absolute accuracy against the distance from the camera to the object, a relative accuracy of 1:17,333 is achieved. This demonstrates highly accurate results meaning that its applicability in close range object 3D modeling is high. If one likes to reduce the cost and the number of camera, it is suggested to utilize at least three cameras aligned in one strip and applying the Triplet Mode in multi-image matching. It can also produce a very accurate and comparable result. However, it might be applied only for object with small relief variation and less occlusion effect.

### 3.4. Other Case Studies: A Human Body and Eight Buddhist Statues

**Figure 16.**
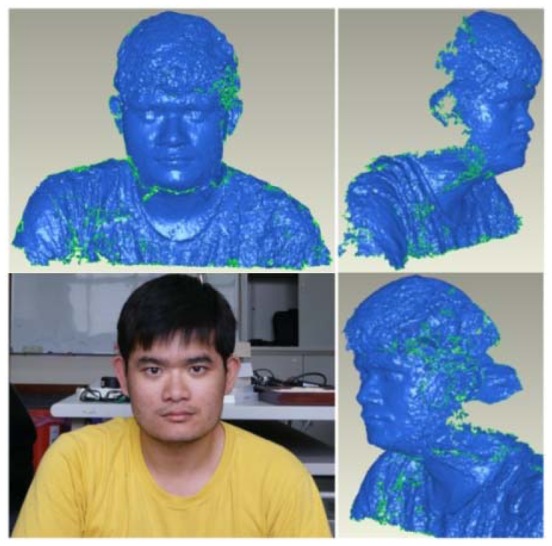
Human model generated by the proposed scheme.

**Figure 17.**
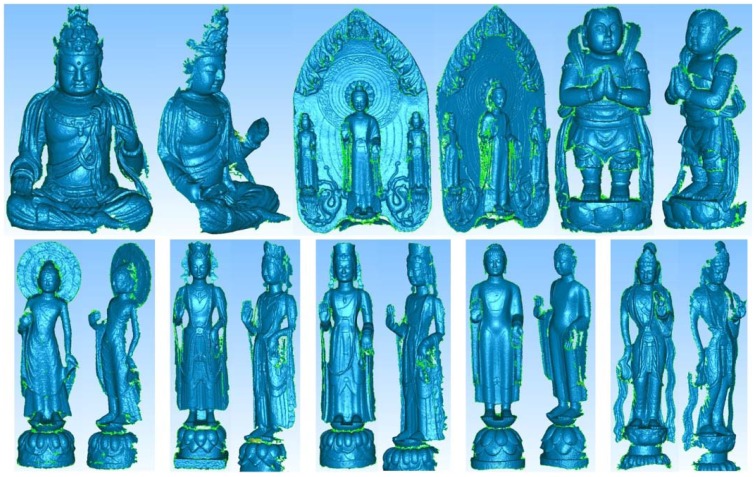
Eight Buddhist statue models generated by the proposed scheme.

In this case study, the proposed close range 3D modeling pipeline is utilized to generate 3D models of a human body and eight Buddhist statues to test its feasibility. The results are illustrated in Figures 16 and 17. For convenient, all of them utilize the 1 × 5 multi-camera configuration because only one metal bar is required. One may observe that the generated 3D models have a higher level of detail in the structure. It is particularly obvious for the Buddhist statues as compared with the human model. This is majorly because the human image has less texture on its surface. The 3D model results of Buddhist statues demonstrate that the proposed scheme would be useful for digital recording of cultural heritage objects. Major museums normally pose hundreds or even thousands of statues, vases, antiques, *etc.*, of similar size. In such cases, it would be very efficient to utilize the proposed scheme by changing the objects while retaining the multi-camera configuration all the time.

## 4. Conclusions

To improve the efficiency of the current image-based 3D modeling approaches, this study proposes an economically advantageous and highly accurate 3D modeling pipeline. The method is particularly efficient when a large quantity of objects with similar size is treated, because no manual operation is needed for photo-triangulation and the quality of the interior and exterior orientation parameters are all the same.

A rotatable camera calibration field with retro-flex code targets is suggested as a means of acquiring suitable calibration images. This can: (i) achieve strong convergent imaging geometry even with limited space; (ii) obtain well distributed tie-points to the whole image frame which is much easier when compared with a fixed calibration field; and (iii) perform fully automatic tie-point recognition and measurement. Experimental results demonstrate its applicability for stability analysis of a camera's internal geometry. We observe the SONY SAL50F14 lens with the automatic initialization functionality which will introduce unstable internal geometry, especially for the principal point coordinates. Thus, it would be better to acquire the calibration images and the target's images before changing the camera's status; otherwise a lens with a fixed focal length and without initialization function is suggested.

For the purpose of quality control during photo-triangulation, particularly when large quantities of objects are treated, a multi-camera framework with an automatic calibration scheme is proposed. An efficient multi-camera calibration scheme is developed using the retro-flex code targets. Depending on indoor or outdoor situations, the code targets can be fixed on a wooden plate or spread on the ground. We can acquire high convergent calibration images by either rotating the wooden plate or the camera's metal bar. Several multi-camera configurations are evaluated using the proposed scheme. The experimental results show that the derived EOPs are accurate and stable, especially for the rotation angles which are very important for close range photogrammetric applications. The major disadvantage of this method is that the combined weight of the DSLR cameras and metal bar(s) is too heavy to be moved. However, this problem can be improved by using lighter consumer grade digital cameras and a more portable metal bar. It should be remembered that in case of the digital recording of a large quantity of antiques or statues stored in a museum, the immobility problem would not be an issue.

Performance analysis of 3D modeling using the proposed multi-camera framework is carried out. A reference model acquired by an ATOS-I 2M scanner is used for comparison. The best result comes from the 2 + 3 configuration that utilizes five cameras with two strips. An absolute accuracy of 0.26 mm is obtained with detailed object surfaces. However, in comparison with the reference model, a smoothing effect along the edge of leafs is unavoidable. This is due to the used area-based image matching technique. Nonetheless, the adopted multi-image matching method can cope with texture-less surface problem and achieve sub-pixel matching accuracy. A relative accuracy of 1:17,333 (comparing the RMSE against the distance from target to the cameras) is achieved. The performance of the proposed method is high. Meanwhile, since the designed scheme can trigger all five cameras simultaneously, the generation of 3D models of dynamic objects is possible. In the case studies, 3D models of the human body and eight Buddhist statues appear to have a high level of detail. The results demonstrate the feasibility of this method for cultural heritage digital documentation particularly for 3D modeling of statues, antiques, *etc.* with a similar size. Since no photo-triangulation or expensive devices are required, the proposed 3D modeling pipeline method would be the most cost-effective approach.

## Figures and Tables

**Table 1.** Significance testing for the determination of additional parameters.

**run**	**items**	**c**	**xp**	**yp**	**K1**	**K2**	**K3**	**P1**	**P2**	**B1**	**B2**
**1**	σ	0.0019	0.0025	0.0015	2.50E-07	1.30E-09	2.00E-12	2.30E-07	1.60E-07	6.40E-06	6.90E-06
	Estimated Value	52.26282	0.120988	-0.05591	5.30E-05	7.37E-09	-1.90E-11	-3.79E-06	-2.64E-06	-1.98E-04	-1.03E-04
	Significant Index	27506.7	48.4	37.3	212.1	5.7	9.5	16.5	16.5	31.0	15.0

**2**	σ	0.0018	0.0026	0.0015	3.60E-08	4.30E-12	4.30E-15	2.40E-07	1.70E-07	6.60E-06	7.20E-06
	Estimated Value	52.25403	0.12224	-0.05537	5.32E-05	0.00E+00	0.00E+00	-3.69E-06	-2.54E-06	-2.01E-04	-1.02E-04
	Significant Index	29030.0	47.0	36.9	1477.3	0.0	0.0	15.4	14.9	30.4	14.1

**3**	σ	0.0018	0.0013	0.0013	3.70E-08	4.40E-12	4.40E-15	4.40E-10	4.40E-10	6.80E-06	7.30E-06
	Estimated Value	52.25684	0.087639	-0.06862	5.31E-05	0.00E+00	0.00E+00	0.00E+00	0.00E+00	-2.02E-04	-1.16E-04
	Significant Index	29031.6	67.4	52.8	1436.1	0.0	0.0	0.0	0.0	29.7	15.8

**run**	**c**	**xp**	**yp**	**K1**	**K2**	**K3**	**P1**	**P2**	**B1**	**B2**	σ_0_ (pixels)	Relative Accuracy	Overall Accuracy
1	■	■	■								2.00	1:6,200	0.3275
2	■	■	■	■							0.21	1:67,200	0.0303
3	■	■	■		■						0.21	1:67,800	0.0300
4	■	■	■	■		■					0.21	1:68,000	0.0299
5	■	■	■	■			■	■			0.21	1:68,600	0.0297
6	■	■	■	■					■	■	0.20	1:70,300	0.0289

**Table 2.** Summary of accuracy analysis results after photo-triangulation for different camera combinations.

**Case No.**	**Camera Combinations**	**σ_0_ (pixels)**	**Relative Accuracy**	**Overall Accuracy (mm)**
1	1,2,3,4,5	0.29	1:114,300	0.011
2	1,2,3,4	0.27	1:102,600	0.012
3	2,3,4	0.26	1:85,700	0.014
4	1,5	0.31	1:73,100	0.017
5	1,4	0.26	1:79,300	0.015
6	2,3	0.27	1:60,600	0.020
7	2,4	0.28	1:69,700	0.018
	Mean	0.28	1:83,614	0.014

**Table 3.** Statistics of accuracy analysis for 3D modeling results.

**Case No**	**# of Strip**	**Camera Locations**	**Multi-Image Matching Mode**	**B/D Range**	**Source of IOP**	**3D Error Analysis (mm)**				**Computation Time in Multi-Image Matching (min)**

						**RMSE**	**Mean**	**Max.**	**Min.**	
1	One	1,4	Stereo	0.56	RT	0.60	−0.014	2.87	−5.00	6
2	One	1,5	Stereo	0.74	RT	0.62	−0.022	3.56	−5.00	6
3	One	2,3	Stereo	0.19	RT	0.30	−0.033	3.50	−2.66	6
4	One	2,4	Stereo	0.38	RT	0.35	−0.027	2.52	−4.66	6
5	Two	1,2,3	Block	0.38	RT	0.28	−0.003	2.65	−4.08	7
6	One	2,3,4	Triplet	0.19∼0.38	RT	0.27	−0.023	3.18	−3.99	18
7	Two	2,3,4	Block	0.38	RT	0.38	−0.023	4.41	−4.18	8
8	One	1 × 5	Block	0.19∼0.74	RT	0.28	0.013	4.81	−4.96	35
9	Two	2 + 3	Block	0.19∼0.74	RT	0.26	−0.002	3.17	−4.81	18
10	One	1 × 5	Block	0.19∼0.74	OTJ	0.59	−0.042	4.43	−5.00	34
11	Two	2 + 3	Block	0.19∼0.74	OTJ	0.49	−0.033	3.80	−5.00	15
